# Injectable Hydrogels Based on Hyperbranched Polymers for Biomedical Applications

**DOI:** 10.1021/cbe.4c00173

**Published:** 2025-02-18

**Authors:** Gaolong Lin, Xiaolin Li, Grzegorz Nowaczyk, Wei Wang

**Affiliations:** † College of Chemical and Biological Engineering, 12377 Zhejiang University, Hangzhou, Zhejiang 310027, China; ‡ ZJU-Hangzhou Global Scientific and Technological Innovation Center, 12377 Zhejiang University, Hangzhou 310027, China; § NanoBioMedical Centre, Adam Mickiewicz University, Wszechnicy Piastowskiej 3, 61-614 Poznan, Poland

**Keywords:** injectable hydrogels, hyperbranched polymers, minimally invasive, degradability, bioadhesion, biomedical applications

## Abstract

Injectable hydrogels (IHs) have garnered significant attention in biomedical applications due to their minimally invasive nature, adaptability, and high degree of customization. However, traditional design methods of IHs have limitations in addressing complex clinical needs, such as precise regulation of the gelation time and mechanical strength within a wide window. Hyperbranched polymers (HBPs), due to their unique highly branched structures and abundant functional sites, can be easily prepared and functionalized to enable decoupled modulation of mechanical properties of IHs and address the clinical challenges of IHs. Our research group developed a library of HBPs via a dynamically controllable polymerization method and built a series of adjustable, controllable, and responsive IHs based on the resulting HBPs. The prepared IHs fed by HBPs demonstrate an adjustable gelation process, a wide-range tuning of mechanical properties, and responsiveness on demand, which show the capabilities in the various biomedical applications. In this review, we summarize the role of HBPs in the gelation process, mechanical properties, self-healing ability, and responsiveness of IHs. However, achieving IHs through HBPs and extending them to a broad range of biomedical applications are still in its infancy. This review provides an overview of IHs fabricated by a variety of multifunctional HBPs, and their biomedical applications in diverse fields are also presented. Meanwhile, we point out the future development of IHs based on HBPs and their potential challenges.

## Graphical abstract

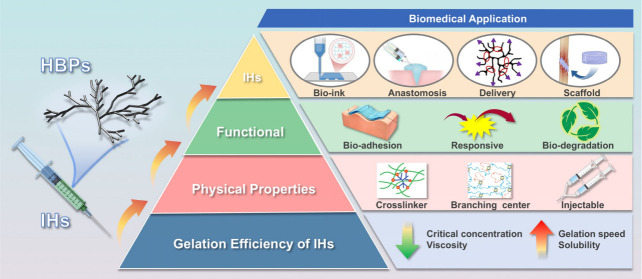

## 1 Introduction

Injectable hydrogels (IHs) can be delivered to various body sites with minimal discomfort while conforming to irregular wound beds, thereby serving as a versatile platform for drug delivery and tissue engineering. Among clinical trials involving bulk hydrogels, injectable variants account for 26%.^1^ IHs play an important role in regenerative medicine by replacing, repairing, or regenerating damaged, defective, or degenerated tissue.^2^ To date, over 30 IH-based products have received approval from the US Food and Drug Administration (FDA) or the European Medicines Agency (EMA). Notable commercial successes include Medtronic’s INFUSE (approximately $750 million) and Endo’s Vantas (approximately $20 million), which have achieved great commercial success.

However, traditional IHs still face many challenges, and it is difficult to fully meet several complex needs: I) the gelation time of IHs is often difficult to decouple, which limits the independent regulation of various properties in design;^3^ II) the mechanical properties of IHs often do not match those of natural tissues, resulting in a poor biointegration;^4–6^ and III) the long-term stability of in vivo applications still needs to be optimized, especially how to maintain their effectiveness in a physiological environment. The precise regulation of the gelation time and mechanical properties of IHs is a tough task by tuning the concentration of linear precursors. A higher concentration of linear precursors will bring a faster gelation process and a stronger mechanical property, but it will lead to a higher viscosity that would decrease the injectable capability and cause premature and uneven gel formation.^7–10^

Attempts to reduce the linear polymer (LP) concentration to improve injectability can compromise gel stability and slow gelation kinetics, potentially resulting in off-target leakage during in situ gelation. In contrast, the highly branched, irregular architecture of branched polymers (BPs) results in reduced chain entanglement, which allows BPs to flow more freely at a lower viscosity and higher solubility. In addition to the above characteristics, BPs also provide abundant terminal groups for functionalization, which makes them suitable for the precise design of materials tailored for specific applications.^11,12^ Although dendritic polymers (DPs) possess highly defined structures and exceptional properties, their complex and time-consuming synthesis limits scalability.^13,14^ Second, hyperbranched polymers (HBPs) are more advantageous due to their relatively straightforward synthesis, enabling large-scale production. Third, HBPs stand out for their excellent biocompatibility due to their abundant active terminal groups and flexible branched structures, which maximize the fixation of monomeric small molecules within the reaction system and reduce the residual toxic monomers in the final product. Fourth, the HBPs can be self-assembled or integrated with other particles (e.g., through nanoparticle filling or cross-linking) allowing for the formation of more sophisticated systems.^3,15^

In the past decade, our research group has developed a library of HBPs via both step growth and chain growth polymerization.^16–21^ Based on these resulting HBPs, a series of IHs was achieved with an adjustable gelation process, a wide-range tuning of mechanical properties, a tractable biodegradation, and an on-demand responsiveness. Those IHs have been explored as biopatches, tissue engineering scaffolds, vehicles to deliver cell and bioactive factors, and so on. In this review, we summarize the experience of the role of HBPs in enhancing the properties of IHs, focusing on their ability to influence gelation processes, mechanical performance, injectability, and functionalization. Through this review, we highlight the unique advantages HBPs offer in overcoming the challenges faced by traditional IHs, underscoring their potential to revolutionize the design of IHs for advanced biomedical applications. The unmet challenges and future developments of IHs based on HBPs will be summarized from a perspective.

## 2 Hyperbranched Polymers

### 2.1 Brief Introduction on HBPs

As indicated in Table 1, compared with LPs, HBPs exhibit a large number of unique properties due to their highly branched structures, such as sparse molecular entanglement, to lead to a low viscosity, a high solubility, and abundant terminal groups for functionalization, which show significant advantages in the construction of IHs. Meanwhile, due to the high density of end groups, the self-assembling nature, and the responsiveness to external stimuli (such as pH, temperature, light, redox conditions, etc.), IHs based on HBPs exhibit numerous advantages over those fabricated by LPs. Although DPs have certain functionalization potential, their synthetic complexity and high rigidity often limit the realization of responsive design.^13,14^ Chauhan and Zhou et al. systematically compared the differences between HBPs and other polymers as amphiphilic self-assembling polymers and found the self-assembly mechanism and unique advantages of HBPs. The special structural characteristics of HBP with a large number of functional groups on the periphery enable it to self-assemble into various structures, such as one-dimensional fibers, tubular bodies, and spherical particles of varying sizes from nanometers to centimeters.^13,22^ From the perspective of application, a significant advantage of HBPs is their ease of synthesis. The synthesis of DPs is laborious and expensive since it involves multiple steps requiring purification between steps. This is in contrast with the one-step (one-pot) synthesis method used in the preparation of HBPs, which makes the scale-up production of these polymers possible and economically acceptable.

**Table 1:**
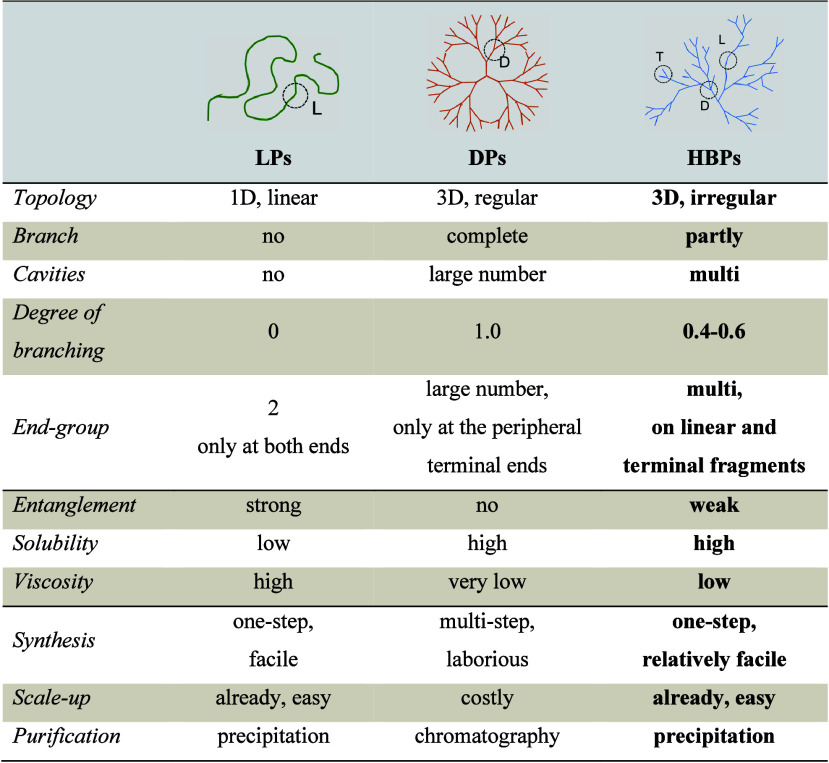
Comparison of LPs, DPs, and HBPs^a^

a “L” is a linear fragment, “D” is a dendritic fragment, and “T” is a terminal fragment.

All of the above properties underscore the versatility of HBPs and highlight their distinct structural and functional advantages over their linear and dendritic counterparts in designing IHs. This unique combination of characteristics not only enhances processability and functionalization but also opens new avenues for smart, responsive materials tailored to complex biomedical applications.

### 2.2 Synthesis of HBPs

Typically, HBPs are synthesized in a one-pot process, which is relatively simple and efficient and is not prone to producing impurities or toxic residues. According to the formation mechanism, the synthesis methods of HBPs can be roughly divided into three categories: step polymerization of complementary monomers, self-condensing vinyl polymerization, and chain growth polymerization of divinyl cross-linkers (Figure 1). This paper focuses on the impact of HBPs in the construction of IHs, so the synthesis is not the main focus of this paper. Readers who need a deeper understanding of the synthesis of HBPs can refer to the previous relevant reviews.^11,23–27^

**Figure 1:**
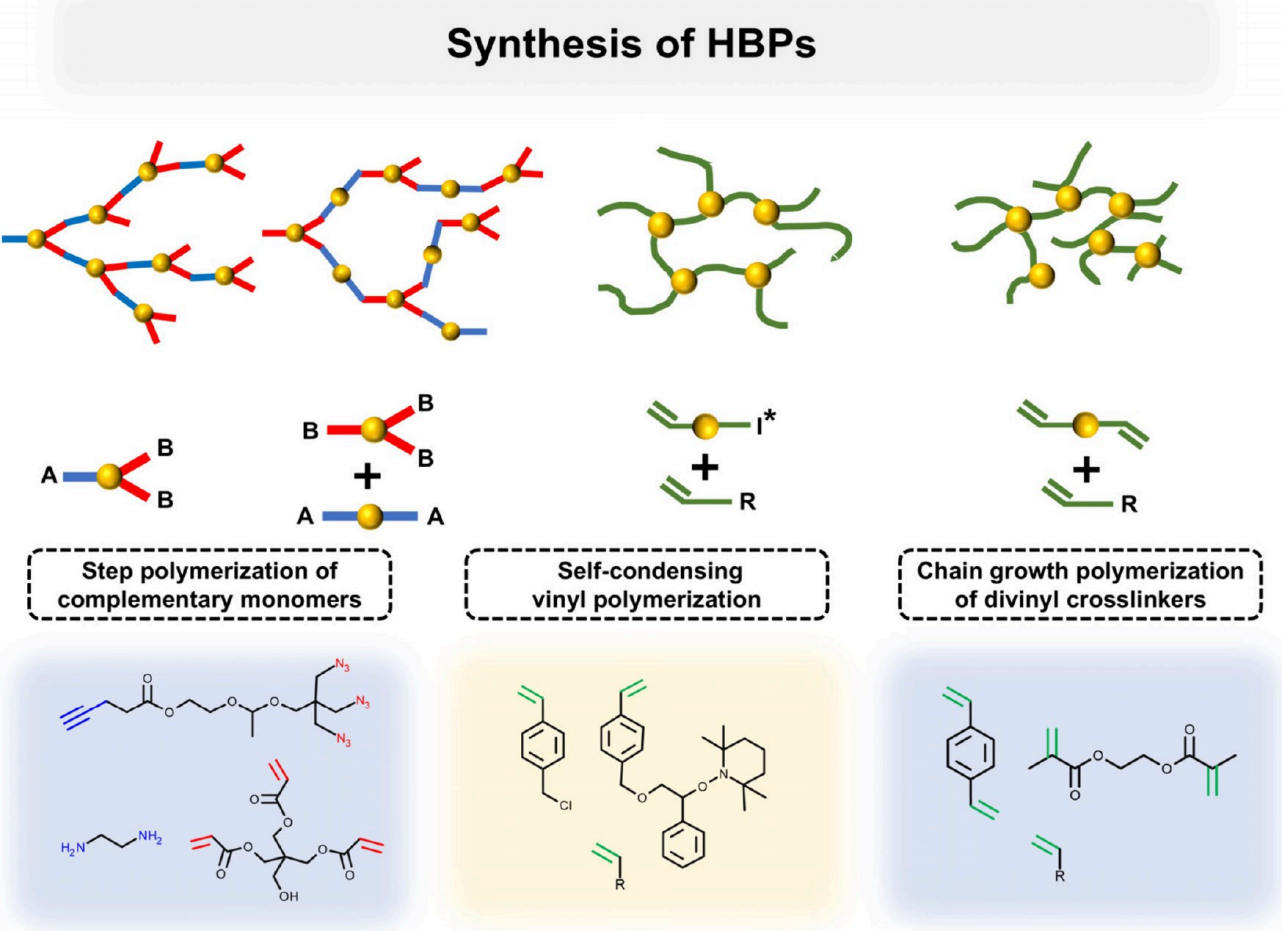
Synthesis of HBPs.

#### 2.2.1 Step Polymerization of Complementary Monomers

Generally, HBPs can be synthesized by a typical step polycondensation of AB_*m*_ (*m* ≥ 2) monomers or the copolymerization of A_*n*_ and B_*m*_ monomers (*n* = 2, *m* ≥ 3), where A and B represent two complementary functional groups that can react with each other but cannot undergo a self-reaction. The advantage of those methods is that the reaction control is flexible, and HBPs with different branching degrees and end-group functionalization can be synthesized according to different designs of functional groups and feed ratios.

The step-growth polycondensation of AB_*m*_ was proposed by Flory et al., which assumes negligible intramolecular reactions. For instance, in the polymerization of the AB_2_ monomer, linear segments form via the reaction of one of the two B groups, while branching occurs when both B groups react with A groups from other monomers. The experimental molecular weight (Mw) distribution of HBPs derived from AB_*x*_ monomers is broader than that of LPs obtained from AB monomers but still narrower than theoretical predictions. Recently, chain-growth polymerization of AB_*m*_ monomers has been successfully achieved by selectively enhancing polymer–monomer interactions while minimizing monomer–monomer reactions. Nonetheless, AB_*m*_-based reactions encounter a significant challenge: these monomers are often difficult to synthesize and are not widely available commercially, complicating their use in polymerization processes.

The polymerization of functionally symmetric monomer pairs, A_*n*_ and B_*m*_ monomers (*n* = 2, *m* ≥ 3), has attracted much attention due to its ability to synthesize HBPs in large quantities simultaneously. The advantage is that the degree of branching (DB) can be better controlled, and the monomers are generally commercially available. Many common chemical reactions, such as Michael addition reaction of amino and olefin double bonds,^28,29^ the formation of hyperbranched polyimides by anhydrides and amines,^30,31^ the formation of hyperbranched aliphatic polyethers by the reaction of hydroxyl groups with epoxides,^32,33^ and the azide–alkyne Huisgen cycloaddition (CuAAC) reaction of azide and alkyne groups,^34,35^ have been used to synthesize HBPs. Among them, the one-step Michael addition method is the most popular one because it is relatively simple, does not require protection/deprotection protocols, does not generate side products that must be removed by further purification steps, and tolerates a wide range of functional groups. In addition, the wide variety of commercially available A_2_ and B_3_ monomers allows for tailoring polymer architectures and provides a more facile preparation route for a variety of HBPs.^36^

#### 2.2.2 Self-Condensing Vinyl Polymerization

The second method to produce HBPs is self-condensing vinyl polymerization (SCVP), which includes both chain growth and stepwise condensation reactions. SCVP was proposed by Frechet in 1995 as a technique for synthesizing AB-type vinyl monomers.^37^ This process utilizes a vinyl monomer pivot, known as the permanent inimer, to initiate polymerization of monomer A*B after activation. The system can incorporate AB_2_, where the starter group A* serves as the A group, while the vinyl group functions as a bifunctional equivalent of B_2_. The initial kinetics is slow during polymerization, and the Mw exhibits exponential growth over time. The ability of each inimer to generate various species complicates the polymer growth mechanism, resulting in highly dispersible HBPs.^38^ The high diversity of monomers and the range of techniques available based on the nature of the starting groups contribute to the widespread use of this method. This method is particularly suitable for the synthesis of HBPs with complex functionalization and can regulate Mw and DB.^24^

#### 2.2.3 Chain Growth Polymerization of Divinyl Cross-Linkers

The third method to prepare HBPs is chain growth polymerization through divinyl cross-linkers, and it can be selected whether to copolymerize with monovinyl monomers. Briefly, a divinyl cross-linker (e.g., divinylbenzene or diene ester) is used to induce cross-linking of polymers. The two double bonds of the cross-linker can act as bridges in the polymerization reaction, connecting different polymer chains to form a hyperbranched structure. This method can also be applied to chemically modify existing polymers (e.g., cross-linking or graft polymerization) to convert them into HBPs.^39–41^ This method is mainly used to prepare HBPs with high cross-linking density. There are a large number of excellent reviews that have summarized them in detail,^11,24,38,42^ so this review will not go into detail.

## 3 Role of HBPs in Properties of IHs

In recent years, HBPs have emerged as a transformative element in the design of IHs, offering a novel approach to address longstanding challenges in the field. The unique architecture of HBPs, characterized by their high branching density and abundant functional groups, opens new avenues for controlling not only the gelation process but also the mechanical performance and injectability of hydrogels. These versatile polymers allow for precise functionalization, enabling the creation of advanced multifunctional hydrogels with applications across various biomedical and therapeutic domains. This section discusses the key role of HBP in improving the performance of IHs, such as increasing gelation efficiency, regulating the physical properties, and achieving multifunctionality.

### 3.1 Gelation Efficiency

HBPs significantly influence the gelation process through molecular design, functional group modification, and cross-linking density regulation. The Mw, DB, functional group distribution, and reactivity with cross-linkers of HBPs determine the gelation rate and network uniformity. A common strategy in step-growth polymerization for constructing polymer networks is “end-linking”, where bifunctional molecules (labeled as A_2_) are joined by multifunctional monomers (labeled as B_*f*_) through reactions between functional groups of A and B. Carothers theorized that gelation of the polymer network occurred when the number-average molar mass of macromolecules in the network formation process approached infinity.^43^ Carothers derived that in step-growth polymerization with equal amounts of A and B groups the critical reaction extent at the gel point, denoted as *P*_c_, was defined as

$$P_{c}=\frac{2}{f_{\mathrm{avg}}},\;\mathrm{where}\;f_{avg}\mathrm{is defined}\;\mathrm{as}\;f_{\mathrm{avg}}=\frac{\sum N_{i}f_{i}}{\sum N_{i}}$$

Here, *N*_i_ is the number of molecules of monomer i with functionality *f*_i_.

Introducing HBPs with high branch functionality (*f*) into the polymer network can increase *f*_avg_, thereby reducing the level of *P*_c_ and promoting gelation. The highly branched structures and multifunctional groups of HBPs can significantly enhance gelation efficiency, allowing for rapid gel formation at lower polymer concentrations. Cai et al. developed an injectable, self-healing hydrogel by combining hyperbranched PEG-based multihydrazide macro-cross-linkers with aldehyde-functionalized hyaluronic acid (HA-CHO). The gel formed within 7 s, attributed to the high density of functional end groups in HB-PEG-HDZ.^44^ Cui et al. reported a fast wet adhesive based on HBPs with a hydrophobic backbone and a hydrophilic adhesive catechol side branch. The branched structure increases the spatial density of components in each independent polymer chain. Therefore, when the HBP adhesive contacts with water, the hydrophobic backbone of HBPs easily aggregates to form a dense microgel network (coacervate) within 10 s. Meanwhile, the coacervate repelled the surrounding water and then adhered to the surface of the object through the catechol group.^45^ Urosev et al. modified the architecture of precursor polymers by introducing a branching structure, where the DB of polymers ranges from 0 to 15%. Increasing the DB of polymers decreased gelation time from 35 to 10 min, in low monomer concentration. An increase in the DB of a precursor polymer enhances the degree of internal cross-linking within the precursor HBP (i.e., more permanent cross-links form within the HBPs themselves). As a result, fewer external cross-links (i.e., cross-links between different highly branched precursor polymers) are needed to reach the gel point, leading to a faster bulk gelation. However, the constrained conformation of the highly branched building blocks significantly limits the gelation kinetics regardless of the polymer concentration. Therefore, at high concentrations, HBPs take even longer to gel than LPs, which has potential advantages in applications where high-weight content gels are required but too fast gelation poses challenges to the gel process^46^ (Figure 2A).

**Figure 2:**
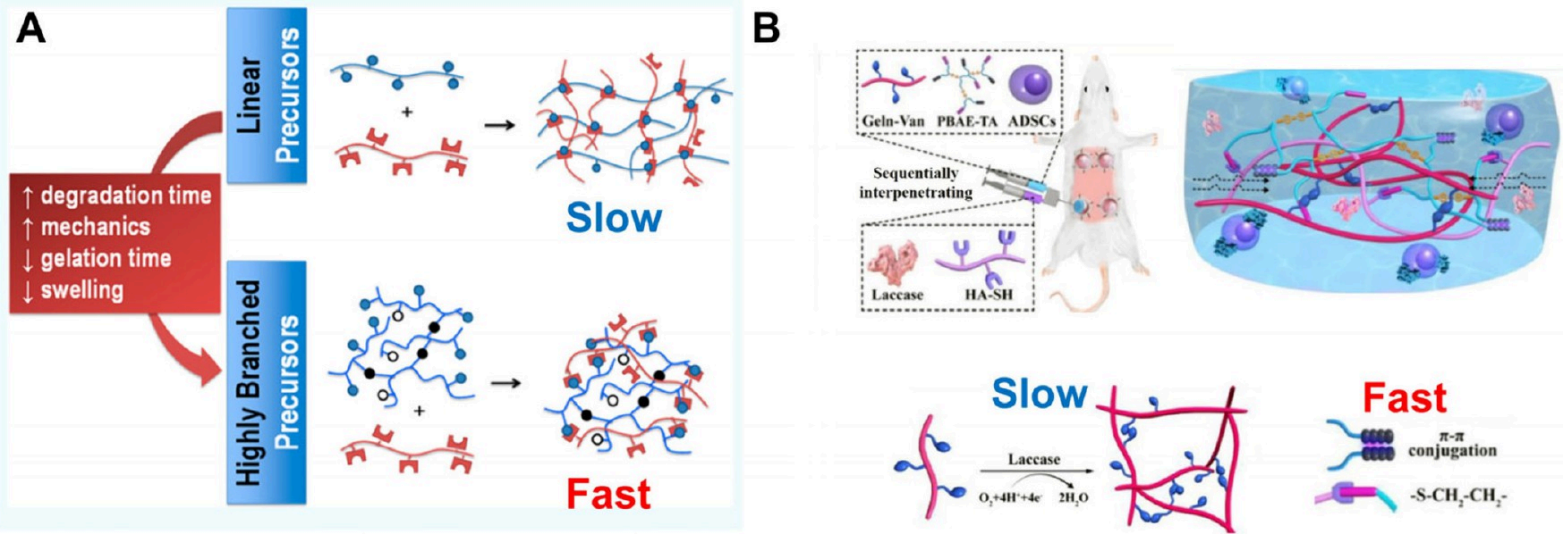
(A) Diagram of HBPs reducing gelation time. (B) Diagram of using HBPs to achieve dual networks with different gelation kinetics. Figure 2A is reproduced with permission from reference 46. Copyright 2019 American Chemical Society. Figure 2B is reproduced with permission from reference 48. Copyright 2020 American Chemical Society.

The formation of gels can be limited by the viscosity or solubility of the precursor solution. HBPs typically exhibit low viscosity and can be easily dispersed in solution, facilitating a uniform molecular distribution, which in turn enhances the homogeneity and structural stability of the gel. This characteristic helps to prevent issues such as phase separation or uneven gel formation during synthesis, thereby ensuring that the final gel exhibits favorable mechanical properties and long-term stability. Furthermore, the low viscosity of HBPs improves flowability during processing, reduces energy consumption during molding, and facilitates superior scalability in large-scale production. Thus, the application of HBPs in gel synthesis presents notable advantages in terms of both processing efficiency and material performance. The fast curing of HBPs is particularly suitable for dual network gels. Wang et al. utilized hyperbranched aminoethyl gelatin with end-grafted catechol (HBGC) to sequentially achieve fast curing (within 10 s) and slow covalent bonds (few minutes) through catechol–Fe^3+^ chelation and HRP/H_2_O_2_, respectively.^47^ Moreover, the three-dimensional network structure of HBPs provides sufficient space for the shuttling of monomers of the second network, ensuring a more stable network.^47^ Jin et al. reported a sequentially interpenetrating dual network based on the combination of a fast “click chemistry” and a slow enzymatic-mediated cross-linking reaction. Laccase could cross-link the vanillin-grafted gelatin (Geln-Van) under O_2_-consuming reactions, in which the progress of cross-linking and oxygen consumption are both based on the enzyme-mediated reaction. However, it causes a paradox between the need for both hypoxic sustainability and fast gelation. To address this issue, hyperbranched poly­(β-amino ester)-tetraaniline (HB-PBAE-TA) was cross-linked with thiolated hyaluronic acid (HA-SH) through a thiol–ene click reaction, enabling the rapid formation of the initial network to ensure structural stability. The structure of HBPs provided sufficient space for the shuttling of monomers and laccase-catalyzed polymerization of the second network. Therefore, the resulting dual network structure exhibited proper network interweaving and a more stable and continuous multinetwork structure^48^ (Figure 2B).

### 3.2 Physical Performance

#### 3.2.1 Injectability

IHs face significant challenges in achieving a balance between injectability, mechanical stability, and self-healing properties, all of which are essential for biomedical translation. The low viscosity and the branched structure of HBP promote shear-thinning behavior, which facilitates smooth injection through narrow-gauge needles.^12^ The structural flexibility of HBPs allows for improved control over the viscoelastic properties of the hydrogel, which plays a critical role in both injectability and retention at the injection site. Li et al. determined the shear-thinning property of hyperbranched polyglycerol-poly­(propylene oxide)-hyperbranched polyglycerol (HPG-PPG-HPG) hydrogels with rheological testing and found that hydrogels based on HBPs were injectable at high gel fraction (Figure 3C–D). Zhang et al. introduced HBPs containing multiple vinyl and catechol groups. This copolymer enables curing reactions with HA-SH after coinjection, achieving a balance between ease of injection and the mechanical strength required for efficient wound treatment.^49^

**Figure 3:**
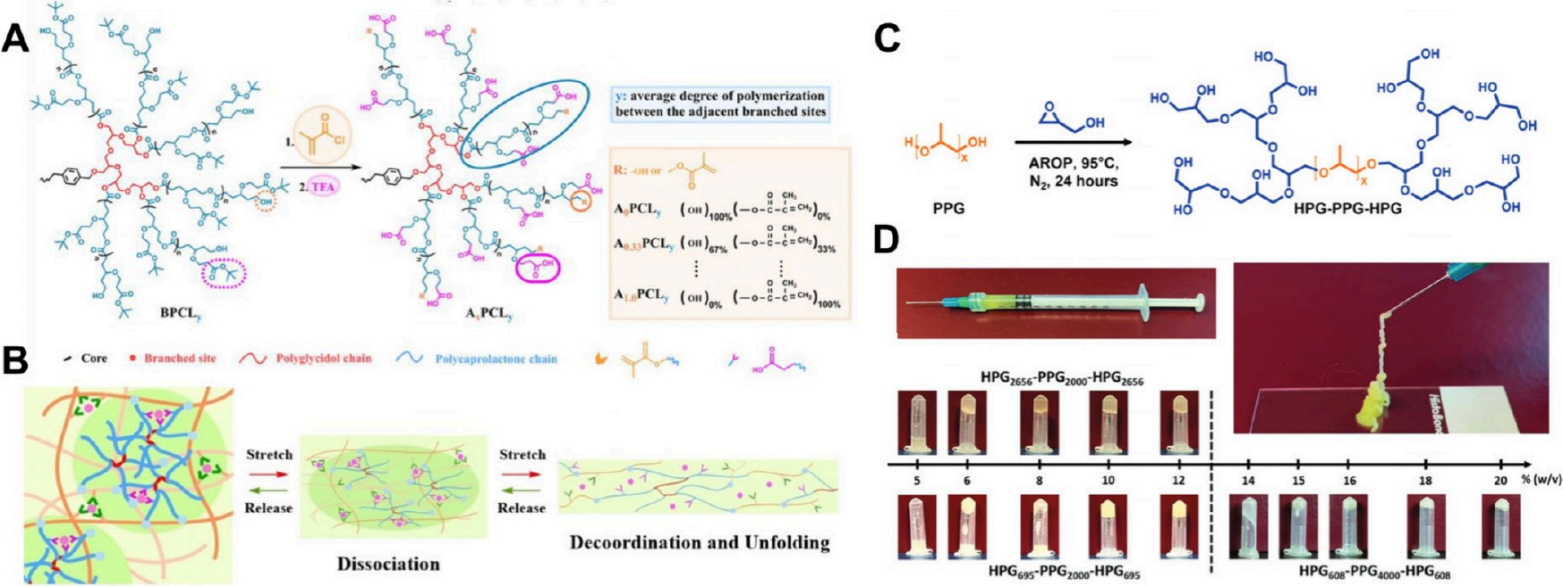
(A,B) Diagram to illustrate the IHs and their energy dissipation mechanism. (C) Diagram to illustrate the synthesis of HPG-PPG-HPG copolymers. (D) Photographs show the injection of hyperbranched HPG-PPG-HPG hydrogel; the numbers represent the Mw of each block. Figure 3A,B are reproduced with permission from ref 54. Copyright 2023 John Wiley & Sons. Figure 3C,D are reproduced with permission from ref 55. Copyright 2023 John Wiley & Sons.

The injectability of hydrogels often relies on dynamic, reversible interactions, such as hydrogen bonding, which, while enabling flow during injection, can compromise postinjection stability due to the inherently weak nature of individual bonds.^50^ When IHs are injected into mechanically dynamic tissue environments, the weak bond of networks results in poor structural integrity after injection, inadequate retention at the target site, and suboptimal biodistribution.^3,51^ The branched structure of HBPs also allows for an increased density of dynamic, reversible interactions such as hydrogen bonds within the hydrogel network. This enhanced interaction density strengthens the adhesive and cohesive forces postinjection, like the role of tannic acid in hydrogen-bonding-based gels.^52^ The free branching of HBPs allows them to drive the recombination of terminal groups through secondary chain relaxation.^53^ Therefore, HBPs can be tailored to form self-healing networks that are restored after deformation. This adaptability enhances the retention of the hydrogel at the injection site and ensures a more uniform distribution within the target tissue, addressing one of the major limitations of conventional injectable biomaterials. Jiang reported a self-healing ionic hydrogel based on carboxyl-functionalized and acryloyl-terminated hyperbranched polycaprolactone (HB-PCL). The hydrophobic domains formed by the spontaneous aggregation of HB-PCL chains and coordination bonds between Fe^3+^ and COO^–^ groups serve as dynamic cross-links. The self-healing ability of the hydrogel was tested in loading–unloading tests, where the hysteresis loops negligibly changed^54^ (Figure 3A,B). Wang et al. constructed IHs with HB-PBAE and HA-SH. The hydrogel can be conveniently injected through a 5^#^ needle onto the myocardial tissue and maintained its shape without any liquid leakage.^21^

Moreover, the low viscosity of HBPs facilitates the enhanced mobility of polymer molecules in solution, preventing diffusion limitations. For in situ gelation after precursor coinjection, a major type of IHs, better diffusion improves the mixing efficiency of the two pregel solutions, enhancing the homogeneity and structural stability of the IHs. The uniform structure provides predictable gel kinetics. In applications, it helps to determine when to inject to obtain sufficient cross-linking strength, which is especially important for progressively gelling IHs.^44^

#### 3.2.2 Network Architecture and Mechanical Strength

IHs often face challenges in achieving precise and wide-ranging control over the mechanical performance, which limits their ability to mimic native tissues effectively. The unique, highly branched architecture of HBPs offers a dense array of reactive sites and diverse functional groups, facilitating multiple cross-linking points and physical entanglements with other components in the hydrogel matrix, which significantly enhances the mechanical strength and structural stability of IHs. Moreover, the branched structures of HBPs influence network diffusion, form dense matrices, and regulate swelling behavior, expanding the methods for controlling the hydrogel properties. This approach combines chemical and physical property modifications and overcomes the limitations of the conventional methods.

Optimizing the network structure of hydrogels is widely recognized as a practical and straightforward approach to enhancing their stiffness.^56,57^ Customizing the branching architecture significantly influences the mechanical strength,^58^ rheological behavior, and processability of polymer hydrogels.^17^ The adjustable rigidity of HBPs branches plays a critical role in forming dense matrices within the hydrogel, effectively preventing excessive swelling.^11,59,60^ For instance, by modulating the DB in HBPs, one can regulate the extent of swelling in the hydrogel network.^61^ Jiang et al. investigated the advantages of hyperbranched topology over linear topology in large cross-linkers. Tensile tests demonstrated that incorporating hyperbranched large cross-linkers into hydrogels improved extensibility by 2–4 times compared to their linear counterparts while maintaining high strength, enhancing the material’s toughness. Microstructural characterization indicated that HBP-based hydrogel possessed a more uniform microphase separation morphology, which should be responsible for the excellent performance^54^ (Figure 4A). The branches of HBPs with adjustable rigidity can form dense matrices and nanoscale pores, which are rarely seen in IHs formed by LPs.^55^ Hong et al. demonstrated that by controlling the Mw and acrylate degree of substitution (DS) of HBP cross-linkers the interactions between monomers or macromers could be finely tuned, producing hydrogels with a wide range of mechanical properties. In this study, HPG was used as a cross-linker for hydrogels, and acrylic HPG (AHPG) with different DS was developed by conjugating the hydroxyl groups on HPG with acrylates to various extents. Although acrylate substitution is the source of the network’s photo-cross-linking ability, the weakening of physical associations between hydrophilic macromers with the increase in hydrophobicity caused by the increase in DS may reduce the extent of the cross-linking reaction. A comparison of the cross-linking strengths at different HPG Mw revealed that longer branches could bind more extensively to the chains and lead to more efficient cross-linking reactions. The effects of three typical monomers, small molecules (e.g., acrylamide), macromers (e.g., poly­(ethylene glycol) monoacrylate (PEGMA)), and proteins (e.g., methacrylate gelatin (MGel)) on gel stiffness were further explored. The results showed that significant branch lengths were required at a given concentration to interact with the macromer chains, enhancing cross-linking. This emphasizes the critical role of the cross-linking polymer’s physical characteristics in shaping hydrogel mechanics.

**Figure 4:**
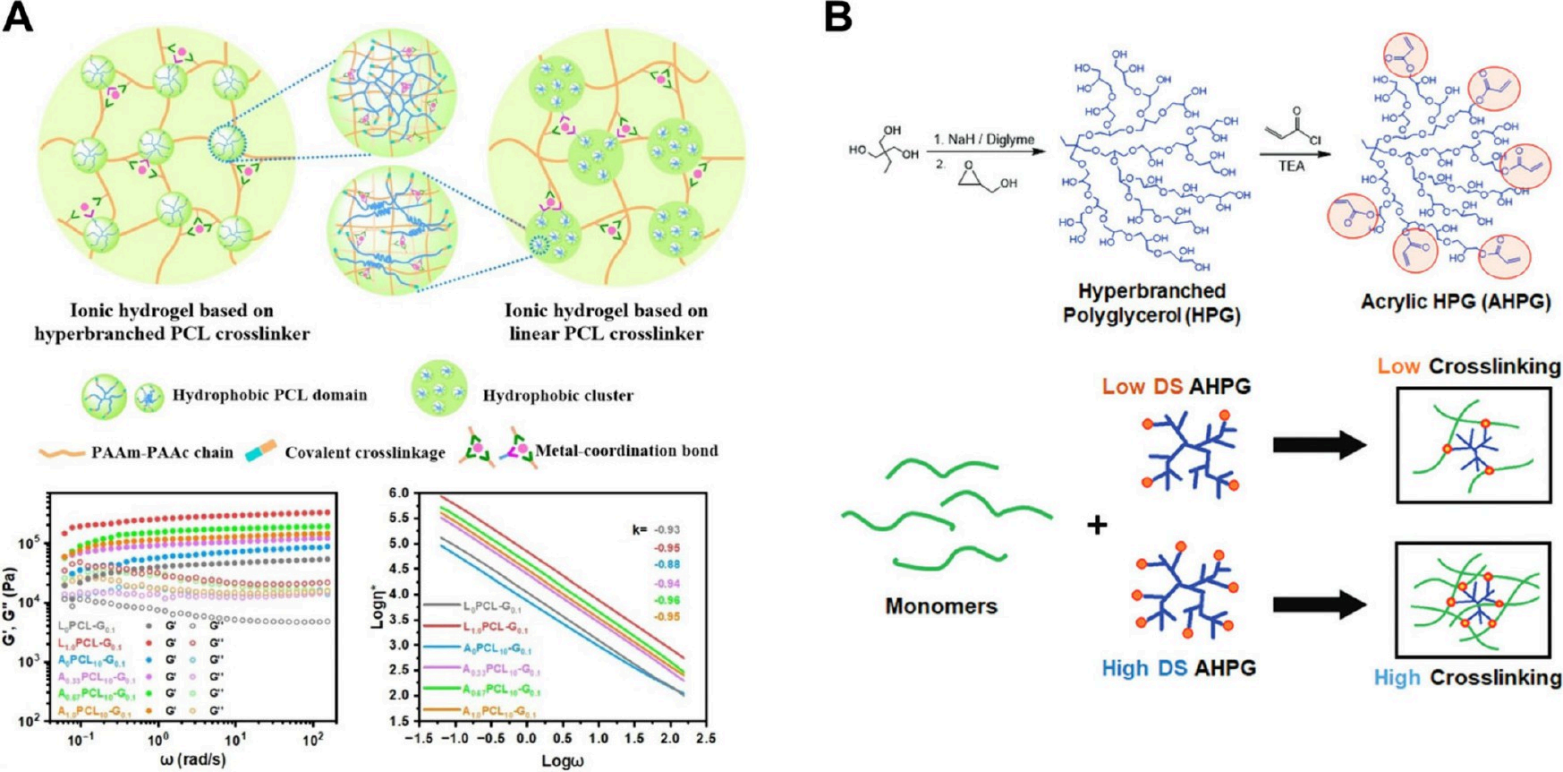
(A) Diagram to illustrate the macromolecular cross-linker based on carboxyl-functionalized and acryloyl-terminated hyperbranched PCL (A_*x*_PCL_*y*_). (B) Synthesis of acrylic-HPG cross-linker, and varying the DS of acrylate in AHPG would allow the control of the cross-linking density of hydrogels. Figure 4A is reproduced with permission from reference 54. Copyright 2023 John Wiley & Sons. Figure 4B is reproduced with permission from reference 64. Copyright 2019 John Wiley & Sons.

A common approach to regulating the mechanical properties of hydrogels involves altering the chemical characteristics of precursor polymers or modifying the quantity and type of cross-linking agents used. These methods can effectively enhance mechanical performance but often result in unintended changes to the hydrogel’s physicochemical properties, leading to a trade-off between mechanical strength and other critical factors, such as biocompatibility, swelling behavior, or degradation rate, and injectability, limiting the flexibility of conventional hydrogels. By adjusting DB or the configuration of the HBP structure, it becomes possible to fine-tune mechanical properties, such as stiffness, elasticity, and toughness, without affecting the desired physicochemical properties of hydrogels.^24,62^ The introduction of HBPs offers a robust solution for the decoupling regulation of hydrogel properties.^46,63^ Adjusting the Mw of HBPs, as opposed to LPs within the same range, was expected to result in minimal changes to the viscoelastic properties of the precursor solution, which is essential for decoupling and modulating the architecture and mechanical properties of the hydrogels without compromising injectability^64^ (Figure 4B). Urosev et al. altered the morphology of HBPs by increasing the DB of HBPs, thereby forming a denser gel network with increased degradation time and stiffness but decreased gelation time and gel swelling.^46^ Even if a small fraction of the total mass of the gel polymer, such as the cross-linker, is replaced with HBPs, it can significantly impact the mechanical properties of the gel network.

### 3.3 Functional Modifications of HBPs for Biomedical Applications

HBPs possess a unique combination of high-density functional groups and facile one-pot synthesis, which lays a robust foundation for their functionalization. The abundance of functional groups within HBPs allows for the precise tuning of their thermal, mechanical, rheological, and solubility properties, offering a versatile toolkit for designing HBPs tailored to a wide range of applications. Functionalization strategies for HBPs typically include terminal modifications, backbone alterations, and hybrid modifications, each providing different levels of control over the behavior of the materials.^65^

The advantages of HBP functionalization can be summarized as practical and safe. From a practical standpoint, the branched structure of HBPs monomer units, combined with the broad spectrum of chemicals available for their synthesis, significantly facilitates the customization of the HBPs. This modular design offers a high degree of flexibility in incorporating diverse functionalities into the polymer, making it possible to achieve targeted properties for specific biomedical or industrial applications.^66^ For instance, Gayen and colleagues developed a series of HBPs with peripheral “clickable” groups, further enhancing the efficiency and versatility of functionalization.^38^ In terms of safety, integrating functional groups into the water-soluble HBPs structure mitigates the risks associated with free functional groups such as uncontrolled reactivity or unintended release within biological systems. This is particularly crucial for applications in sensitive environments such as drug delivery or tissue engineering, where the stability and safety of the material are paramount. Incorporating functional groups directly into the HBPs matrix reduces potential toxicity and ensures that the desired functional properties are stably retained within the system.^16^ Thus, the inherent design flexibility and safety profile of HBPs make them ideal platforms for creating advanced functionalized hydrogels.

#### 3.3.1 Bioadhesion

The retention and biodistribution of injected hydrogels at the site of administration are often overlooked, primarily influenced by bioadhesion and biodegradation.^3^ Adhesive performance is governed by two forces: adhesion and cohesion.^67^ Adhesion pertains to intermolecular interactions that bind the adhesive to the tissue surface, while cohesion relates to the internal structural integrity of the adhesive. Both forces contribute to the overall adhesive strength through energy dissipation. Therefore, designing bioadhesive gels requires balancing the strength and distribution of adhesion and cohesion. HBPs can achieve controllable spatial structure and binding site distribution to tailor the adhesion and cohesion,^14^ and their rich end groups can adapt to a variety of binding modes.

HBPs provide a highly branched structure containing multiple branches, exhibiting a relatively unique internal structure and exposing functional groups on their surface, just like folded proteins, which can be used as scaffolds for multivalent anchoring and cross-linking. Xie et al. synthesized HB-PBAE through a Michael addition reaction between DOPA, PEG diacrylate (PEGDA), and pentaerythritol triacrylate (Figure 5A). HB-PBAE provides a highly branched scaffold, which imitates the chemical structure of mussel adhesive proteins (MAPs), the key natural substance that enables mussel byssus to adhere to wet surfaces in seawater.^19^ HBPs combined with catechol groups provide a more effective mimic of MAPs, further advancing their applications for bioadhesion. Wang et al. achieved rapid bioadhesion by coinjection of catechol-modified HBPs with Fe^3+^ solution and oxidant (Figure 5B). Based on the premise that HBPs functionalized with catechol groups may offer broader application potential, a series of such functionalized HBPs has been developed, and their possible uses have been investigated.^16,19,45,47,49^ While numerous cationic polymers have been created for surface modification and adhesive purposes, the mainstream approach remains the addition of catechol groups and their derivatives. It is hoped that future research will introduce new methods, expanding the horizons for the synthesis of bioadhesive HBPs.

**Figure 5:**
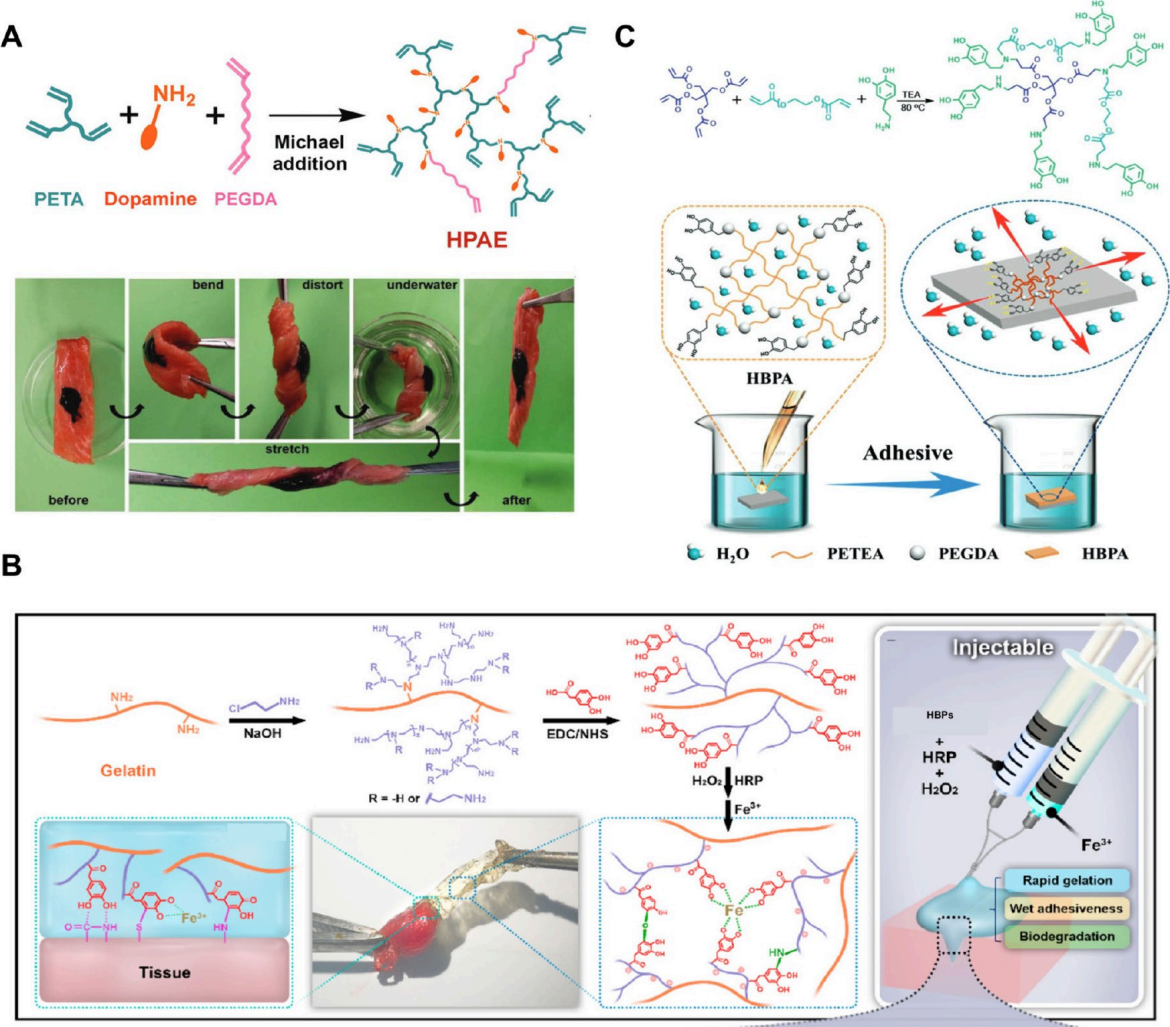
(A) Diagram to illustrate bioadhesive of HB-PBAE hydrogel on porcine myocardium tissue. (B) Diagram to illustrate the development and applications of catechol-modified HBPs for bioadhesion. (C) Diagram of water-triggered strong underwater bioinspired adhesion of HBPs and the underlying adhesion mechanism. Figure 5A is reproduced with permission from reference 16. Copyright 2018 John Wiley & Sons. Figure 5B is reproduced with permission from reference 47. Copyright 2022 Elsevier. Figure 5C is reproduced with permission from reference 45. Copyright 2019 John Wiley & Sons.

The presence of hydrated water on surfaces has been shown to impair adhesion under wet and underwater conditions. Consequently, removing water from the adhesive interface and improving the wettability of adhesive on the substrate are crucial for ensuring a robust underwater adhesion. Underwater adhesion of coacervate can be achieved by phase separation, which increases the hydrophobicity and displaces water molecules on the adherent surface. Several complex coacervate adhesives with linear structures have been reported; however, their coacervation in water typically requires external triggers, such as temperature, pH, or ionic strength. It may be due to the low density of the adhesive group and complex topology. Instead, Cui et al. synthesized HBPs adhesive (HBPA) with a hyperbranched hydrophobic backbone and high-density adhesive hydrophilic catechol side chains, where the hydrophobic backbone rapidly self-aggregates upon contact with water, forming coacervates that expose catechol groups, resulting in strong adhesion to various materials across different environments.^45^ Furthermore, owing to the branched structure and numerous functional chain terminals of HBPs, the long alkylamine can be introduced into this modular hyperbranched architecture to achieve rapid hemostasis without affecting the adhesion^45^ (Figure 5C).

#### 3.3.2 Biodegradation

Efficient biodegradation is essential to avoid adverse biological reactions, reduce tissue rejection, and promote reconstruction of the organization. Especially in tissue engineering and medical implants, biodegradation is a key factor in ensuring that materials can promote cell proliferation and tissue regeneration while avoiding toxic or inflammatory responses. Hydrophilic-leading HBPs degrade faster than hydrophobic ones, which benefits their biodegradation.^16,68^ At present, a large number of biocompatible polymers such as HPG, poly­(ethylene oxide), and sugar derivatives and biodegradable polymers such as hyperbranched polyesters, polyphosphates, peptides, etc., have been carefully designed and widely used in the biological field.^65^ In addition, HBPs can also be functionalized with degradable or biologically responsive motifs to improve their degradability and biosafety.

A variety of different chemistries and multifragment designs of branched monomer units that can be used in the synthesis of branched polymers benefit the incorporation of biologically responsive motifs and degradable bonds into polymer structures. Disulfide bonds are cleavable under reductive conditions or photolysis, making them a commonly employed bond as a degradable moiety^20^ (Figure 6A). Cuneo et al. summarized a series of examples of degradable units incorporated into hyperbranched polymer backbones in their review.^24^ Branched linkers, including acetal,^69–73^ ester bond,^74–77^ and dithioester, are degradable due to their hydrolysis in vivo. Cystamine has a disulfide bond and can also be polymerized with acrylate-based polymers through the Michael addition reaction. Due to its ease of use, it is often used as the branching center of HBPs. HB-PBAE was biodegradable with 3,3′-dithiobis (butanoic hydrazide) (DTP) serving as the branched center and PEGDA serving as cross-linker (Figure 6B). Hydrogel synthesized from Michael addition of PEGDA with cystamine was found to be ROS-sensitive. Meanwhile, the drug in hydrogel is gradually released as the hydrogel undergoes biodegradation.^21^

**Figure 6:**
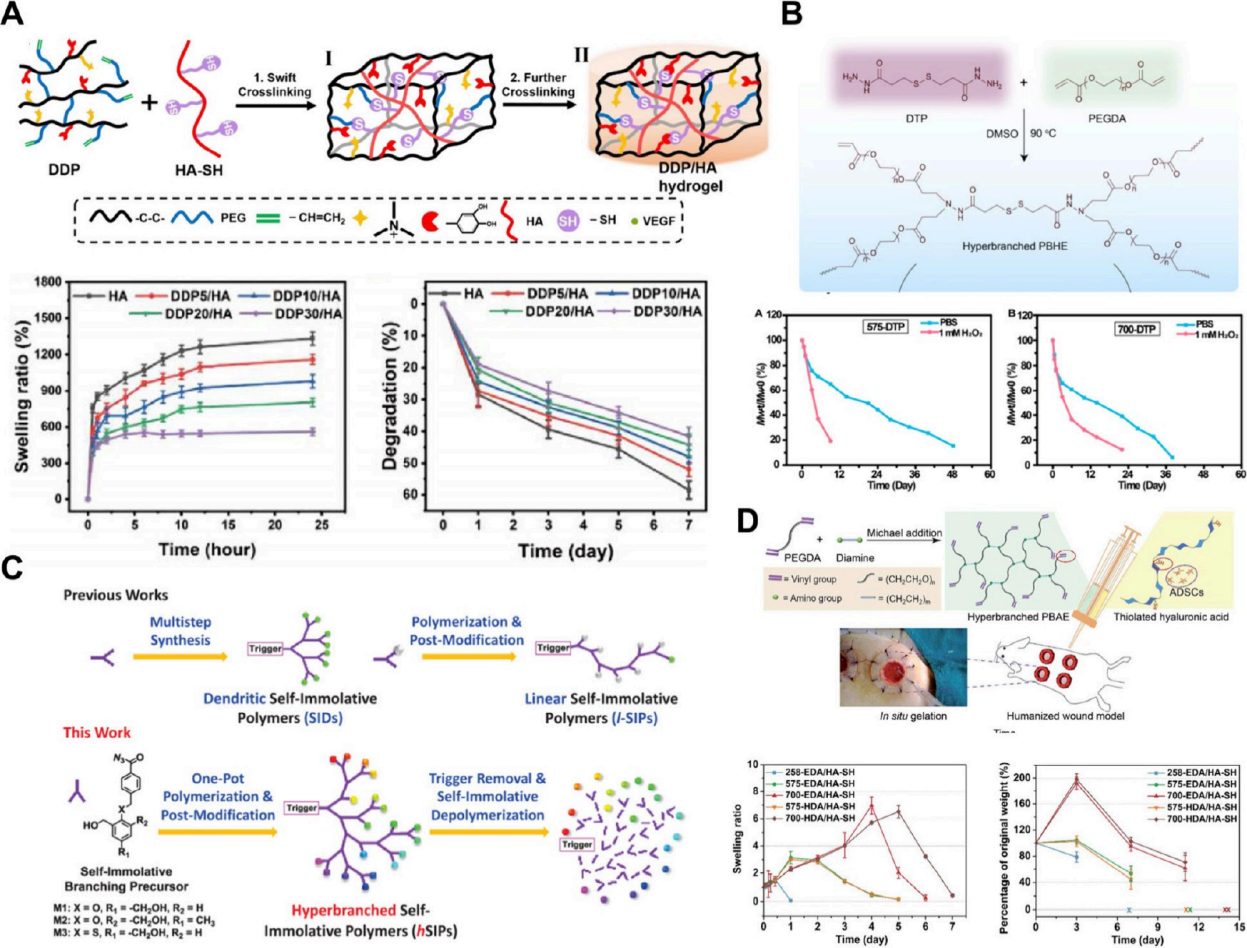
(A) Diagram to illustrate the generation and degradation of injectable hyperbranched hydrogel with disulfide bonds. (B) Diagram to illustrate the generation and degradation of HB-PBAE, where DTP serves as a H_2_O_2_-sensitive branched center. (C) Diagram to illustrate the degradation of hSIPs. (D) Diagram to illustrate the generation and degradation of HP-PBAE. Figure 6A is reproduced with permission from reference 49. Copyright 2024 Elsevier. Figure 6B is reproduced with permission from reference 20. Copyright 2018 American Chemical Society. Figure 6C is reproduced with permission from reference 80. Copyright 2015 American Chemical Society. Figure 6D is reproduced with permission from reference 79. Copyright 2018 Royal Society of Chemistry.

When used as biomaterials, different polymers can achieve similar performance through chemical design, including instantaneous adhesion strength and mechanical strength, but they often show large differences in biodegradability. In the research of Bochynska et al., while evaluating the physical chemistry and adhesive performance of the three-arm and override embedded segment cluster adhesive, they also compared their degradation performance in the body and outside. When used as an adhesive for meniscus tears, networks based on the HBPs show a faster mass loss compared to networks prepared from the three-armed polymers in vivo.^78^ This stimulates in-depth research on the influence of the branched structure on the degradation rate. It is worth noting that simply introducing a degradable part is not enough for biosafety as degradation products may also cause adverse reactions in organisms. Xu et al. developed degradable hydrogels by HB-PBAE, which are made from diamine (ethylenediamine) (EDA), PEG-dialdehyde (PEGDA), and HA-SH. The multiple ester groups in the backbone promote hydrolysis under physiological conditions, producing noncytotoxic amino acids and diols as degradation byproducts^79^ (Figure 6D). To make the polymers decompose more completely, researchers designed self-immolating polymers (SIPs) to construct biosafe materials. Liu et al. reported a series of hyperbranched self-immolated polymers (hSIPs). Upon stimuli-triggered single cleavage of capping moieties at the focal point and chain terminal, self-immolated polymners undergo spontaneous domino-like radial fragmentation^80^ (Figure 6C).

In addition, there are some cases where the degradation rate needs to be appropriately slowed, especially for hydrogels of natural macromolecules. When HBPs are introduced into these natural hydrogels, the compact, branched molecular structure and abundant terminal active groups of the HBPs effectively increase the cross-linking density of the resulting hydrogels. The increased cross-linking density enhances mechanical strength and structural stability, preventing the rapid degradation typically seen in natural hydrogels such as HA and chondroitin sulfate (CS). Pure CS scaffolds degrade quickly, posing a challenge for regenerating neo-tissue similar to natural articular cartilage. To address this, Li et al. selected PEG-based HBPs as structural constituent materials for gelation due to their low immunogenicity, superior mechanical properties, and long-term stability in vivo. The CS-SH/HB-PEG hydrogel scaffolds were created via a thiol–ene reaction, offering rapid gelation, excellent mechanical strength, and slower degradation. This combination of CS and hyperbranched multifunctional PEG copolymer synergistically promotes cartilage repair.^81^ Goodarzi et al. incorporated branched polymers to delay the degradation of gelatin cryogel by improving the physical cross-linking and crystallinity.^82^ The team of Ziyi Yu synthesized a series of biodegradable hydrogels comprising HA-SH and hyperbranched poly­(β-hydrazide esters) (HB-PBHE).^83–88^ HB-PBHEs uphold the structural integrity of the hydrogels, preventing the rapid enzyme-mediated degradation of the pure HA hydrogels. The compact branched molecular architecture of HBPBHEs, combined with their numerous terminal double bonds, provides both low toxicity and high reactivity with HA-SH, significantly enhancing the cross-linking density of the resulting HA hydrogels. Notably, compared to hydrogels cross-linked with conventional agents like butanediol diglycidyl ether (BDDE), the hydrogels in this study demonstrate considerably slower degradation rates. Moreover, incorporating disulfide groups in HB-PBHEs improves the biocompatibility of the material as it is degradable in both in vivo and in vitro environments.^83^

#### 3.3.3 Stimuli-Responsiveness

Stimuli-responsive hydrogels are characterized by their ability to respond to stimuli of the tissue microenvironment such as changes in temperature,^89,90^ pH,^91–93^ ROS,^20,85,94^ and so on. These properties can be exploited for stimuli-triggered drug release,^95^ nanomedicine,^96^ shape memory,^97,98^ and tissue engineering.^19,20,47,85,92,94^ Stimuli-responsive HBPs have gained increased attention in recent years. Their unique globular, void-containing topological structure, featuring numerous terminal functional groups and branches, results in lower solution or melt viscosity and better solubility, making them highly suitable for advanced stimuli-responsive systems.^42^

In the research of Cui et al., the large hydrophobic main chain, the small hydrophilic tail, and the branching center are polymerized into a HBPs network through the Michael addition reaction. Due to the existence of the branched structure, the number of hydrophobic or hydrophilic groups in a single polymer is significantly increased. At the same time, the long hydrophobic chains are distributed in the chain segments at intervals so that their aggregation is rarely interfered with by steric hindrance, and the same is true for the hydrophilic groups. Therefore, with water as the triggering condition, spontaneous aggregation of hydrophobic groups occurs inside HBPs, displacing surrounding water molecules.^45^ Moreover, the change in the hydrophobic group also triggers the rearrangement of the hydrophilic tail. According to the chemical characteristics of the hydrophilic tail, more response behaviors could be exhibited, including chemical adhesion affected by the density of functional groups, fluorescence quenching and enhancement affected by the distance of functional groups, etc.^99,100^ Lu et al. prepared a microenvironment-responsive hydrogel based on hyperbranched poly­(amino acid) (HPTTG) to avoid using catheterization and real-time image guidance angiography in embolization therapy. The pH of the tumor microenvironment is 6.7–7.2 because of a high rate of anaerobic glycolysis in solid tumors due to lactate accumulation. HPTTG hydrogel underwent a sol-to-gel phase transition with decreasing pH. The pH value of the transition was controlled by adjusting the ratio of acidic amino acids in copolymers.^92^ Jin et al. reported ROS-responsive hydrogel nanoparticles (HBPTK-Ce6 NPs) with light-triggered size reduction based on self-assembly of an amphiphilic hyperbranched polyphosphoester containing thioketal units and photosensitizers, Chlorin e6. The nanoparticles have an initial average diameter of ∼210 nm for stability in blood circulation. Upon 660 nm laser irradiation on tumor tissues, the nanoparticles effectively generate ROS, which cleaves the thioketal, therefore sequentially reducing the size of nanoparticles, which facilitates more efficient tumor penetration^101^ (Figure 7A).

**Figure 7:**
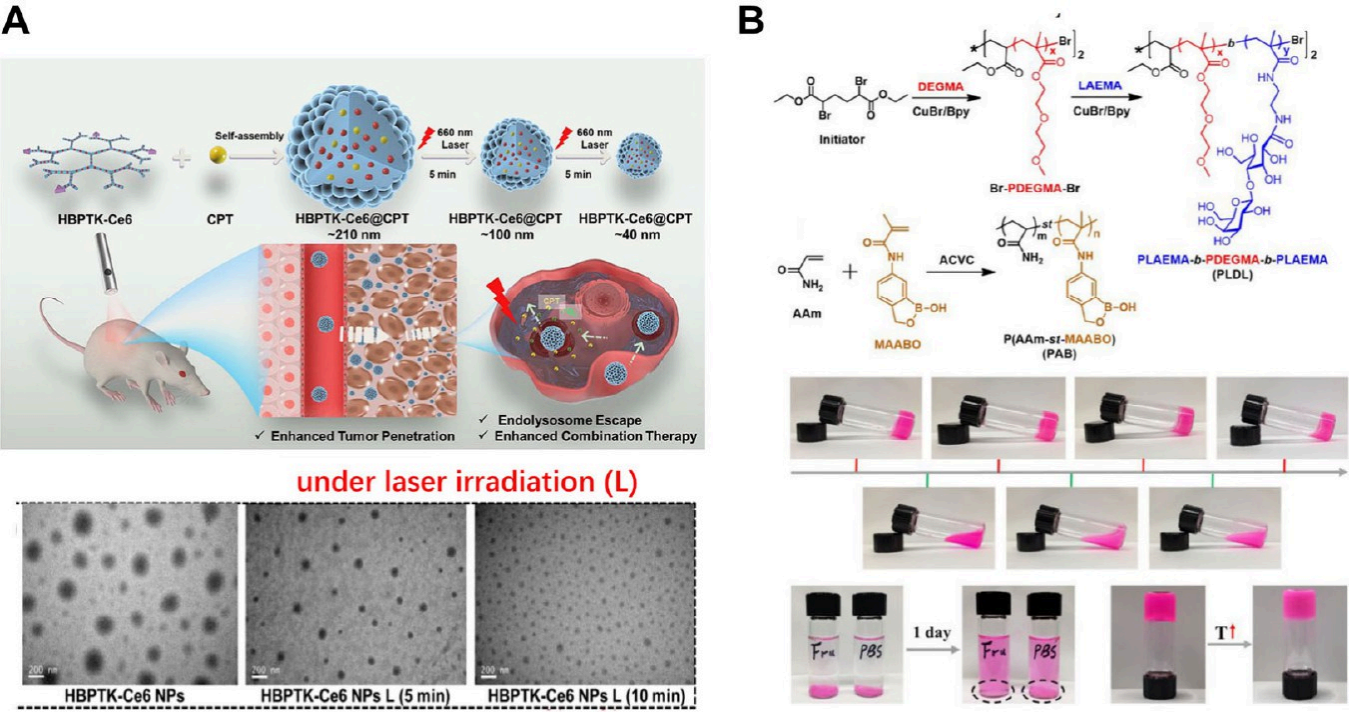
(A) Diagram to illustrate light-triggered ROS-responsive hydrogel nanoparticles baed on hyperbranched polyphosphoester (HBPTK-Ce6). (B) Diagram to illustrate the synthetic route of branched PLDL copolymer and its responsiveness toward pH, sugar, and temperature. Figure 7A is reproduced with permission from reference^101^. Copyright 2019 Elsevier. Figure 7B is reproduced with permission from reference 102. Copyright 2019 American Chemical Society.

Given the complex physiological environment of the human body, there is a demand for multiresponsive hydrogels capable of performing various functions in response to different signals. Therefore, the integrated design of polymer chains and cross-linking sites in HBPs can facilitate the development of multiresponsive, self-healing hydrogels.^102^ Chen et al. developed an ABA triblock copolymer featuring a thermoresponsive central block and hydrophilic glycopolymer chains at both termini. Hydrogels were subsequently formed by blending this triblock copolymer with a linear hydrophilic copolymer that contained benzoxazole groups. The formation of dynamic covalent bonds occurred through interaction between benzoxazole groups and sugar hydroxyls. Hydrogels cross-linked by boronic esters have inherent pH and diol responsiveness. Therefore, the resulting hydrogels exhibited multiresponsiveness to temperature, pH, and sugar^95,102^ (Figure 7B). In summary, multiresponsive HBPs can be formed by aggregating multiple responsive fragments in chains of a single HBP. However, attention should be paid to the mutual interference of different responsive units in the hyperbranched network structure. In addition, most current response phenomena of HBPs are based on the formation and dissociation of dynamic chemical bonds,^58,103,104^ and few attempts have been made to construct complex systems with responsive changes in structural conformation.

## 4 Biomedical Application of IHs Based on HBPs

Due to their abundant active end groups and inherent branched structures, HBPs can be developed into multifunctional platforms for biomedical applications in multiple scenarios. First, based on their excellent biocompatibility and injectability, IHs fed by HBPs can be used in bioinks to precisely print biocompatible three-dimensional structures. Combined with end-group functionalization, adaptive closure and adhesion can be achieved in complex wounds. In addition, the multilevel structure inside HBPs gives it excellent material loading capacity and heterogeneous structure, forming a gel system with hierarchical pores and multiphase networks. It enhances the functions of the gel in the delivery of cells and drug. Finally, by integrating multiple aspects of performance, HBPs can be used as tissue engineering scaffolds and implanted in vivo to promote tissue repair under greater mechanical loads and more complex biological environments.

### 4.1 Bioink

Bioprinting is an advanced technique for constructing functional tissues or organs through the precise spatial positioning and assembly of living cells and biomaterials.^64^ IHs fed by HBPs are ideal bioinks due to their excellent biocompatibility, tunable mechanical properties, and rapid gelation ability. Sigen et al. developed a printable instant gelation IHs system based on a designed hyperbranched PEG-based multihydrazide (HB-PEG-HDZ) as a macro cross-linker and HA-CHO. Due to the high functional group density of HB-PEG-HDZ, the hydrogel forms instantly upon mixing the two-component solutions and minimizes the residual toxic ingredients, which ensure its biocompatibility. The reversible cross-linking between hydrazide and aldehyde groups imparts shear-thinning and self-healing properties to the hydrogel and prevents damage to cells during extrusion. The resulting IHs protect cells during printing and enhance the survival of transplanted cells^105^ (Figure 8A).

**Figure 8:**
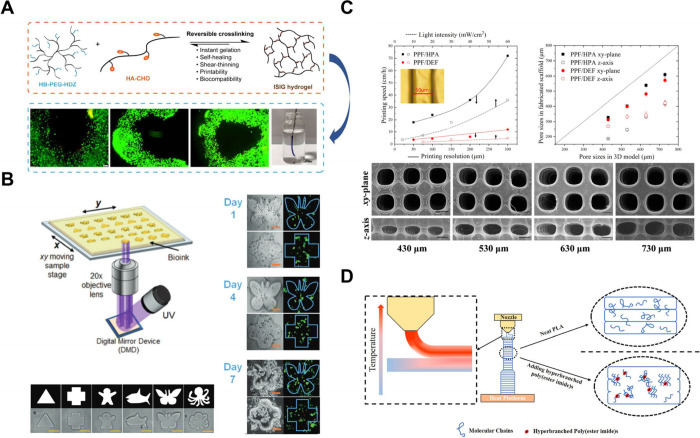
(A) Diagram to illustrate the synthetic route of IHs and improved survival of transplanted cells in it. (B) Diagram to illustrate cell-laden IHs cured by UV light and viability of encapsulated cells during 7 days. (C) Diagram to illustrate the 3D printing of PPF scaffolds based on HPA instead of linear DEF. The printing speed, the pore size, and the morphology of printed gels of different formulations. (D) Diagram to illustrate that HBPs improve the interlayer adhesion of 3D-printed parts during printing. Figure 8A is reproduced with permission from reference 105. Copyright 2020 American Chemical Society. Figure 8B is reproduced with permission from reference 64. Copyright 2019 John Wiley and Sons. Figure 8C is reproduced with permission from reference 107. Copyright 2021 Elsevier. Figure 8D is reproduced with permission from reference 109. Copyright 2024 Elsevier.

Hong et al. reported an IHs bioink based on AHPG. The AHPG IHs were made from acrylic-HPG. By controlling the Mw and acrylate DS of the HBPs cross-linker, the interaction between the cross-linker and various types of monomers can be changed, thereby controlling the mechanical properties of the gel and its precursor solutions. Owing to the branched structure of AHPG, the mechanical properties of the hydrogel cross-linked with AHPG can be controlled by the acrylate DS even without changing the monomer concentration, which will also ensure that the viscoelastic properties of various precursor solutions remain largely constant, greatly improving the stability and consistency of the printing procedure. A digital light processing (DLP)-based projection printing system was utilized to fabricate AHPG-linked hydrogel arrays in various shapes with micrometer-scale resolution (microgels), showcasing their potential as mechanically tunable bioinks for the development of microtissue constructs. The cure of AHPG bioink was triggered by micropatterned UV light reflected by the microscopic lens. The structure of AHPG hydrogel also provides protection and a suitable microenvironment for the encapsulating species and therefore can be used as cell-laden bioink^64^ (Figure 8B). Zhou et al. reported a peptide-branched PEG-reinforced bioink (HC-PDN), which contained branched PEG (PDN) grafted with peptide dendrimers and hyaluronic acid modified with cysteine (HC). The introduction of PDN facilitated the grafting of ample functional groups and enhanced thiol–ene-induced cross-linking, enabling HC-PDN to withstand greater compressive loads (with strains increasing from 42% to 150%). The resulting HC-PDN is reversible and flexible, making it suitable for extrusion 3D printing. Adjusting the PDN level influences the mechanical properties of the gel, which, in turn, affects the activity of cells seeded within it. By inoculating different hepatocytes into HC-PDN bioink or GelMA and printing them in layers, a bionic liver tissue model with heterogeneity can be constructed. The printed bionic liver tissue model exhibits structural and functional characteristics resembling those of liver tissue in vivo.^106^

Li et al. used hyperbranched polyester acrylate (HPA) instead of linear diethyl fumarate (DEF) for the 3D printing of poly­(propylene fumarate) (PPF) scaffolds. Blending HPA with PPF decreased the viscosity of the solution and accelerated the process of photo-cross-linking. At a high printing resolution of 50 μm, replacing DEF with HPA can increase the printing speed from 3.6 to 18 cm/h. The PPF/HPA scaffolds demonstrated improved stiffness and toughness and reduced shrinkage compared to their linear counterparts (PPF/DEF). The introduction of hyperbranched HPA greatly optimizes printing performance^107^ (Figure 8C). Wheeler et al. demonstrated the potential of high-Mw hyperbranched methacrylate polymers (HBMA) for inkjet printing. HBMA exhibited significantly higher maximum printable concentrations in comparison to their linear counterparts, enabling faster printing speeds. Moreover, HBMA showed superior resistance to flow-induced Mw degradation. This resilience arises from their ability to rapidly maintain a thermodynamically stable Gaussian coil conformation during jetting. Consequently, the contraction flow in the print head does not fully transmit to the polymer, effectively suppressing degradation.^108^ In addition, adding even a small amount of HBPs to conventional polymer matrices can significantly enhance printing performance. For instance, blending hyperbranched poly­(ester imide)­s (HBPEIs) into a poly­(lactic acid) (PLA) matrix improves the interlayer adhesion of 3D-printed parts^109^ (Figure 8D). Similarly, incorporating fully aromatic photosensitive hyperbranched polyaryletherketone into UV-curable resin systems enhances thermal performance, mechanical properties, and dielectric properties.^110^ Incorporating hyperbranched triethoxysilane reagent (HPASi) that contains multiple supramolecular hydrogen bonding into dynamic covalent imine/Diels–Alder network facilitated secondary cross-linking, and the resulting hydrogels presented a strengthened self-healing and temperature-responsive shape memory effect.^97^ HBPs can also provide unique hydrophilic/hydrophobic environments due to their branches and internal voids. Zhao et al. discuss the design and development of a 3D-printable hydrophobic silicone ink using a hyperbranched poly­(urethane acrylate) (PUA) monomer. The PUA acts as a stabilizing agent, allowing the incorporation of high amounts of hydrophobic monomer without phase separation.^111^

### 4.2 Anastomosis of Complex Wounds

The injectability of IHs allows them to be used for the minimally invasive delivery and sealing of cracked tissue. HBPs endow the gel with high injectability and adaptive ability to irregular shapes and dynamic wounds. Therefore, IHs fed by HBPs can be used for the anastomosis of complex wounds. Liang et al. employed two-step Michael addition reactions to synthesize HB-PBAE with pyrrole end-capping. To improve the biocompatibility of the hydrogel patch, gelatin was added. The resulting physical cross-linked hydrogel could be easily painted and bonded tightly onto the rough myocardium tissue. The adhesion lasted for more than 4 weeks; it remains closely attached to the myocardium tissue during the degradation process in vivo; and the degradation products have no obvious biological toxicity. Meanwhile, pyrrole nanoparticles were generated in situ in the gel, which endow the gel patch with the ability of electrical conduction^16^ (Figure 9A). Dong et al. synthesized HBPs IHs using HA and HB-PEGDA for the regeneration of burn injury. It undergoes rapid in situ gelation upon contact with wounds, forming a conformable dressing that fits the wound shape.^112^ Barua et al. reported a highly biocompatible surgical sealant based on an s-triazine-based hyperbranched epoxy and a poly­(amido amine) hardener. The addition of hyperbranched epoxy can significantly improve toughness, and its surface functional groups and high reactivity help form a rigid network, making it easy to process. Hyperbranched epoxy can be degraded under in vivo conditions without producing toxicity. In addition, hyperbranched epoxy also has inherent antibacterial ability, effectively preventing wound infection.^113^ HBPs themselves can also enhance platelet aggregation and activation through functionalization with zwitterionic sulfabetaine and cationic quaternary ammonium ligands in a concentration- and positive charge density-dependent manner, promoting hemostasis.^114^

**Figure 9:**
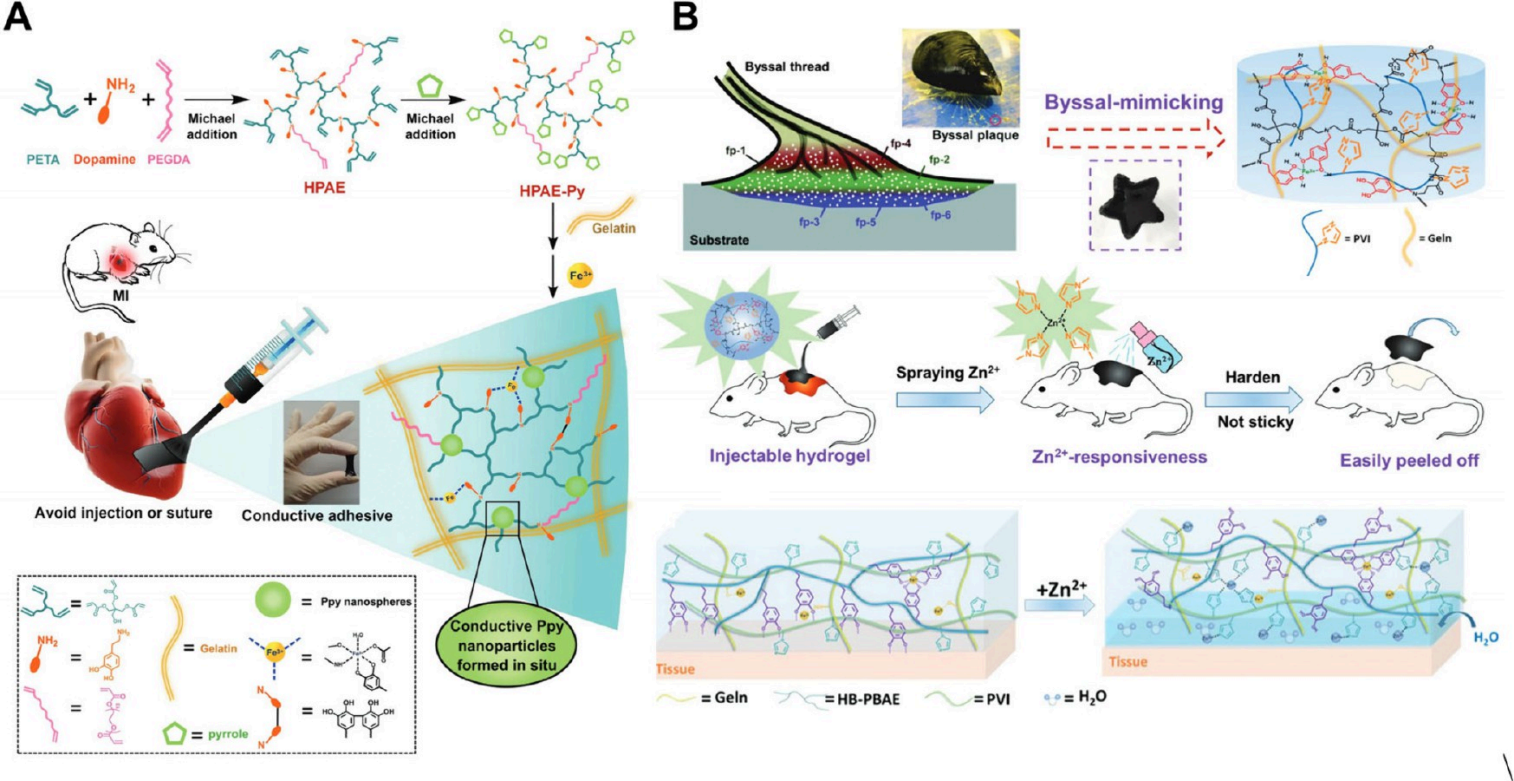
(A) Diagram to illustrate the synthetic route of HPAE-Py and a paintable HPAE-Py hydrogel patch for porcine myocardium tissue, exhibiting outstanding bioadhesive characteristics. (B) Diagram to illustrate the bioinspired adhesive hydrogel based on a hyperbranched DOPA structure and illustrating the wound dressing change depending on responsiveness toward zinc ions. Figure 9A is reproduced with permission from reference 16. Copyright 2018 John Wiley and Sons. Figure 9B is reproduced with permission from reference 19. Copyright 2020 Royal Society of Chemistry.

Zheng et al. developed an injectable adhesive by doping porous particles (MBC@CMS) with dopamine-functionalized hyperbranched polymers (HPDs). The branched and amphiphilic structure of HPDs caused the hydrophobic chains to aggregate upon contact with water, increasing the exposure of surface catechol groups and thereby enhancing the wet adhesion of the composite adhesive. Remarkably, the adhesive maintained its adhesion properties even under running water and water immersion, effectively sealing water outlets.^115^ Xie et al. synthesized IHs based on HB-PBAE as a replaceable wound dressing to ease pain during dressing changes. HB-PBAE has catechol and imidazole groups. Initially, multicatechol groups on the HB-PBAE might be oxidized by Fe^3+^ or complexed with Fe^3+^, both of which contributed to the strong wet adhesive property. Imidazole groups on HB-PBAE can be complexed with Zn^2+^ and respond within tens of seconds. It sharply increases the water worming in the hydrogel, subsequently promoting surface wettability of the hydrogel. The formation of hydration layers reduces the close contact between adhesive polymers and tissue surfaces, disrupting both bidentate hydrogen bonds and hydrophobic interactions between the hydrogels and the surfaces. Spraying of the Zn ion solution resulted in a rapid decrease in adhesive strength and a dramatic increase in mechanical properties, thus being able to serve as a trigger for a response, enabling the change of wound dressings in a mild, noninvasive manner^19^ (Figure 9B). Luo et al. used hyperbranched polyglycidyl ether (HBPE) to prepare multifunctional IHs with a fast gelation time, self-healing ability, and repeatable adhesion. HBPE facilitated a biocompositing strategy that incorporated conductive MXene sheets and graphene. The resulting composite IHs were mechanically flexible, antibacterial, electroactive, bioadhesive, self-healable, and hemostatic and were used as a flexible wounded treatment–health monitoring bioelectronic implant.^116^

### 4.3 Delivery Systems for Cells and Drugs

Due to their three-dimensional spherical structure and ease of synthesis, HBPs have garnered significant attention in the development of drug delivery systems. Li et al. synthesized HPG-PPG-HPG polymersomes using an oil-in-water emulsion, followed by centrifugation to obtain hydrogels with aqueous cavities. The cavities encapsulated hydrophilic drugs through rehydration with a drug solution. The drug encapsulation efficiency and drug release rate from hydrogels were determined by the void size and hydration capacity, while the void size of the hydrogels was decided by the fraction and Mw of the HPG block. IHs formed by HBPs with compact and tunable networks address the challenges of efficient encapsulation and sustained release of small hydrophilic molecules, which are often limited in traditional hydrogel systems due to their large mesh size and high water content.^55^ Pan et al. reported a binary hydrogel precursor system based on HA-SH and hyperbranched PEGDA. These two components were produced into uniform hydrogel microparticles (≈130 μm) by using microfluidic technology to encapsulate miR-29a-abundant exosomes, providing the necessary bioactivity. Then, the hydrogel microparticles were collected to form granular hydrogels with excellent injectability, which was verified by measuring the injection pressure during a stable continuous hydrogel extrusion using a 3D extrusion printer. The granular hydrogels were injected into the fracture site of the bone by a 26 G syringe^86^ (Figure 10A). Zou et al. synthesized IHs through aniline tetramer grafted hyperbranched epoxy macromer (AT-EHBPE) and HA-SH. The IHs encapsulated exosomes via an epoxy/thiol “click” reaction. The exosomes were labeled with a fluorescent probe and visualized under IVIS. The hydrogels significantly prolonged the retention of exosomes in vivo.^117^ Wang et al. constructed ROS-sensitive IHs by synthesizing HB-PBAE and HA-SH, and the drug loading method involved combining tanshinone IIA (TIIA) nanoparticles (NPs) with a polydopamine (PDA) layer to form TIIA@PDA NPs, which were then encapsulated within the hydrogel through chemical cross-linking between thiol groups and quinone groups on the PDA. The incorporation of TIIA@PDA NPs significantly improved the mechanical properties and drug-loading capacity of the hydrogel. The IHs exhibited slow degradation behavior in vivo and markedly enhanced cardiac function, reduced infarct size, and suppressed the expression of inflammatory factors^21^ (Figure 10B).

**Figure 10:**
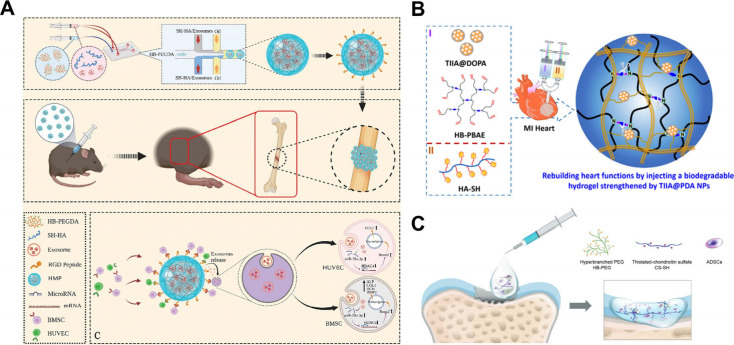
(A) Diagram to illustrate the synthetic route of injectable granular hydrogels encapsulating exosomes and their application. (B) Diagram showing the IHs incorporating nanoparticles for drug delivery. (C) Diagram showing the IHs based on hyperbranched multifunctional HB-PEG and CS, encapsulating ADSCs. Figure 10A is reproduced with permission from reference 86. Copyright 2023 John Wiley and Sons. Figure 10B is reproduced with permission from reference 21. Copyright 2019 American Chemical Society. Figure 10C is reproduced with permission from reference 81. Copyright 2021 Royal Society of Chemistry.

Fan et al. combined hyperbranched copolymer hydrogels (HBDLDs) with dendritic nanogels (DNGs) to achieve codelivery of antibiotics, where the hydrophilic drug novobiocin sodium salt (NB) is entrapped within the hydrophilic hydrogel, while the hydrophobic antibiotic ciprofloxacin (CIP) is encapsulated within hydrophobic cores of DNGs. The hybrid hydrogels enable the quick release of NB and prolonged release of CIP.^118^ Liang et al. prepared injectable nanocomposite hydrogels based on carboxymethyl chitosan and polylactic acid-HPG. Owing to the abundance of functional groups in HBPs, they not only facilitate the formation of an IHs network and the encapsulation of drug molecules but also enable in situ tissue adhesion through reversible Schiff base bonds. This versatility achieved a three-in-one functionality of adhesion, drug delivery, and retention, and solved current intraperitoneal drug delivery systems face issues: rapid drug clearance from lymphatic drainage, heterogeneous drug distribution, and uncontrolled release of therapeutic agents into the peritoneal cavity.^119^ HBPs have also been developed as a responsive delivery carrier. For example, Zhao et al. used electroactive hyperbranched polyamidoamine (EHP) as the cross-linking center of hydrogels to make multiple stimulus-responsive drug delivery systems. Due to the unique molecular architecture and dramatic conformational transition of EHP toward voltage, hydrogels of EHP exhibited fascinating pH-stimulus drug release behavior in both acidic and alkalescent environments.^120^

The synthesis of HBPs does not rely on organic solvents, where the reaction substrate is safe and green; therefore, the cytotoxicity of HBPs hydrogels is relatively lower than hydrogels made from traditional synthetic polymers. Many studies take the porous network structure of HBPs IHs as a three-dimensional template for cell delivery.^44,64,81,90,105,121–130^ Li et al. produced HBPs based on the combination of CS and hyperbranched multifunctional poly­(ethene glycol) copolymer (HB-PEG) via the thiol–ene reaction. Hybrid hydrogel scaffolds have demonstrated strong mechanical properties, appropriate porosity, and excellent biocompatibility. The rat adipose-derived mesenchymal stem cells (ADSCs) seeded in the hydrogels presented improved chondrogenesis and great cell viability. Moreover, due to the well-documented anti-inflammatory activities of CS, the IHs scaffolds reduced the inflammatory response of stem cells. The hydrogel integrating with ADSCs was injected into certain sites for cartilage regeneration^81^ (Figure 10C). Dong et al. developed an in situ-formed hydrogel system to deliver adipose-derived stem cells for the treatment of burn wounds, which comprised a hyperbranched PEGDA polymer and a commercially available HA-SH. HBPs enable tunable swelling and mechanical properties of the hydrogel, which fit stem cell culture.^112^ When cells come into contact with matrix materials, they interact with the matrix through adhesion molecules (such as integrins), begin to adhere, and gradually spread.^131^ Changes in the cell morphology trigger a series of signal transduction events that directly affect the physiological activity of cells. Therefore, when using HBPs to construct IHs for delivering cells, appropriate functionalization should be considered to promote cell adhesion and subsequent specific spreading in a three-dimensional structure. RGD (Arg-Gly-Asp) peptides were incorporated into HBPs hydrogel to alter the cellular morphology and enhance cell proliferation, affecting tissue remodeling.^86,112^ In addition to adding a cell adhesion motif, it is also possible to directly activate the reactive groups on the surface of HBPs to improve affinity toward the cell. Zhang et al. synthesized HBPs IHs composed of HA-SH and HB-PBHE. The surface of hydrogel could be partially degraded by enzymes, where HBPs were unaffected, maintaining structural integrity and providing exposed reactive sites for cell adhesion.^83^

### 4.4 Scaffolds for Tissue Engineering

By adjusting the physical parameters of HBPs, such as Mw and DS of reactive functional groups, the mechanical properties of the resulting hydrogels can be controlled in a wide range.^54,64^ Through precise design, scaffolds can provide mechanical strength, elastic modulus, and pore structure similar to native tissues, ensuring that they can withstand physiological loads and work synergistically with surrounding tissues in the in vivo environment. Moreover, biomimetic materials produce less stress and impact on surrounding tissues during and after implantation, reducing the risk of secondary injury. IHs are mainly used as tissue engineering scaffolds in osteoarthritis,^132–134^ cartilage repair,^135,136^ wound fixing,^137,138^ and nerve regeneration.^4^ Bochynska et al. evaluated the adhesive properties of novel biodegradable hyperbranched block polymeric adhesives serving as a treatment for meniscus tears. The building blocks of the hyperbranched adhesive were PEG, trimethylene carbonate (TMC), citric acid (CA), and hexamethylene diisocyanate (HDI). The HBPs adhesives have sufficient adhesive strength (66–88 kPa) to meniscus tissue after curing and have tensile properties in the same range as those of the human meniscus. The values of the elastic modulus (*E*) and the maximum tensile stress (σ_max_) were in the same range as those of the human meniscus.^78^ Grinstaff et al. designed IHs adhesives prepared from highly branched polymers to replace or supplement sutures in the repair of corneal wounds. Highly branched lysine-cysteine dendrons with thiol and amine were cross-linked to PEGDA to produce IHs adhesives, which are transparent, pliable, and soft.^139^ Wu et al. prepared hydrogels that can simultaneously mimic the structure and function of the skin. The hydrogels were generated via polymerizing functionalized extracellular vesicles as a hyperbranched cross-linker. The obtained compartmentalized cross-linked networks exhibit enhanced mechanical strength compared with conventional divinyl monomer-cross-linked hydrogels due to the dissipation of stretching energy caused by vesicle deformation.^140^

Liu et al. produced IHs based on poly­(propylene fumarate) (PPF) and a hyperbranched PCL as the cross-linker core. The IHs showed enhanced biocompatibility and low heat generation during cross-linking and were injected into vertebral bodies of the rabbit spine and can be monitored by X-ray imaging after incorporating zirconium dioxide (ZrO_2_) powder^141^ (Figure 11A). Yang et al. prepared IHs using a novel thiol/thioester dual-functionalized hyperbranched polypeptide and maleimide-functionalized polysarcosine under biologically benign conditions. The IHs encapsulated mesenchymal stem cells (MSCs) were filled with osteochondral defects as osteochondral substitutes. It exhibits suitable biodegradability, excellent biocompatibility, and low immunogenicity and possesses an immunomodulatory role for defective cartilage^142^ (Figure 11B). Guo et al. produced an injectable nanocomposite hydrogel based on *N*-hydroxysuccinimide-functionalized hyperbranched PEG (NHS-PEG), proteins, and MgO nanoparticles (MgO NPs). NHS-PEG acts as a cross-linking center, and the network was reinforced by MgO NPs, which led to more amide bond formation. Those reactions endowed IHs with enhanced mechanical properties and the ability to instantaneously solidify and stabilize gel under harsh environments, such as moisture and bleeding. The synthesized IHs promote mandible regeneration in osteonecrosis of the jaw by inducing the formation of vessels, activating osteoprogenitor cells, and creating an anti-inflammatory microenvironment. Furthermore, the hydrogel’s enhanced osteogenic properties and implantation feasibility were demonstrated in the mandible and iliac crest defects created in minipigs, respectively^143^ (Figure 11C).

**Figure 11:**
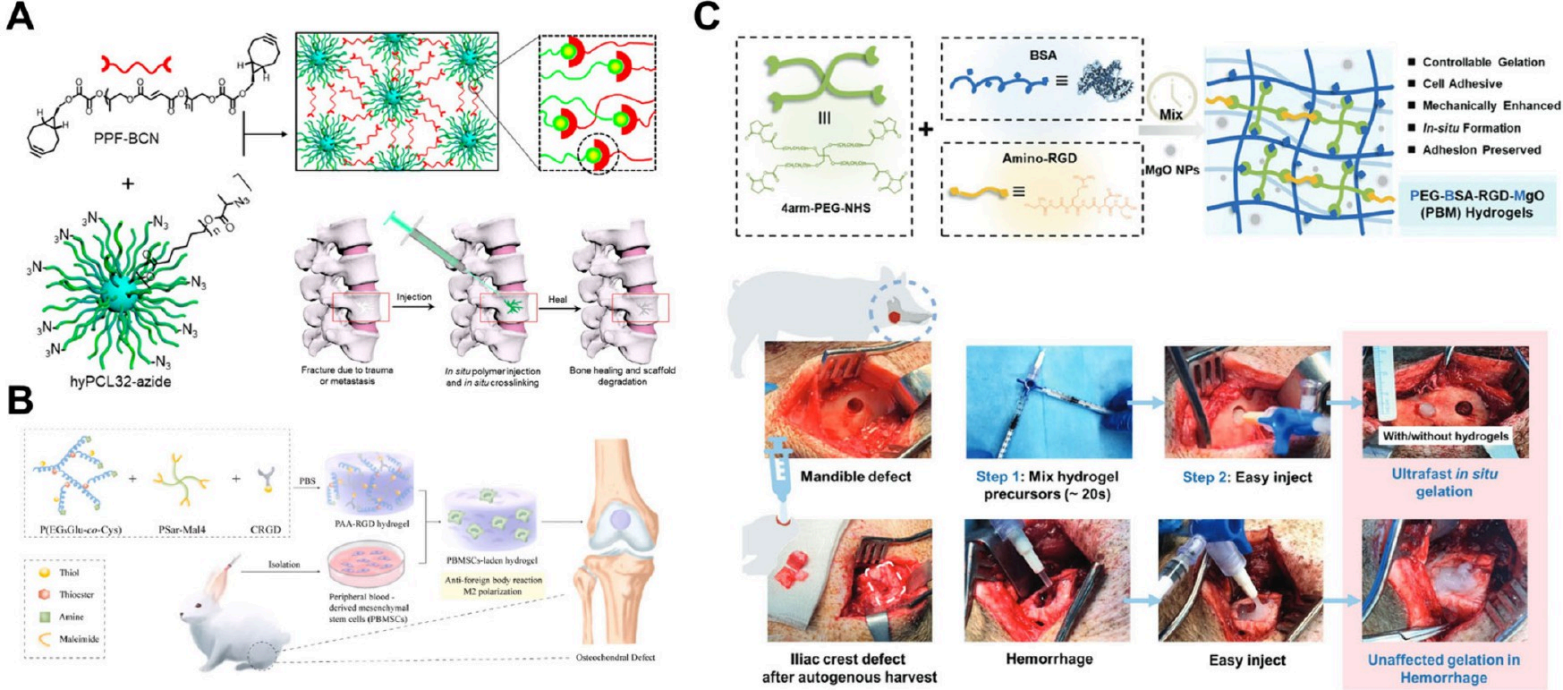
(A) Diagram to illustrate the synthetic route of IHs based on PPF and a hyperbranched PCL and its application as a scaffold for vertebral body repair. (B) Diagram to illustrate the synthetic route of cell-laden scaffold and its transplantation into defect cartilage. (C) Diagram to illustrate the synthetic route of IHs based on hyperbranched NHS-PEG, proteins, and MgO NPs. The IHs were injected into the defect site of the mandible and iliac crest defects. Figure 11A is reproduced with permission from reference 141. Copyright 2019 American Chemical Society. Figure 11B is reproduced with permission from reference 142. Copyright 2023 Elsevier. Figure 11C is reproduced with permission from reference 143. Copyright 2024 John Wiley and Sons.

## 5 Conclusion and Perspective

In HBPs, the highly branched structure prevents segment entanglement while providing abundant terminal groups, offering several advantages for constructing IHs: I) a balance between injectability, mechanical stability, and self-healing properties; II) the formation of structurally uniform multicomponent interpenetrating networks; III) the ability to decoupling regulate the mechanical properties of the hydrogel and achieve efficient gelation across a broad range of monomer concentrations; and IV) minimized toxicity of residual monomers. Besides, HBPs have significant advantages for the functionalization of IHs, especially from the perspectives of safety and practicality. HBPs endow IHs with multifunctionality, including bioadhesion, biodegradation, and stimulus responsiveness, enabling tailored biomedical applications. Despite undeniable progress, challenges and limitations remain. While the synthesis of HBPs is relatively simple, it compromises structural accuracy and may lead to increased batch variability, and the inherent structural uncertainty of HBPs complicates the study of structure–performance relationships. These underscore the need for comprehensive structure–performance investigations, robust predictive models, and bench-to-bedside validation. Given the remarkable clinical success of IHs and the unique advantages that HBPs offer in their construction, the field is on the brink of groundbreaking advancements that will further transform biomedicine.
